# Key community eye health messages

**Published:** 2017-11-11

## Babies born before 36 weeks (preterm) are at risk of retinopathy of prematurity (ROP)

**Figure:**
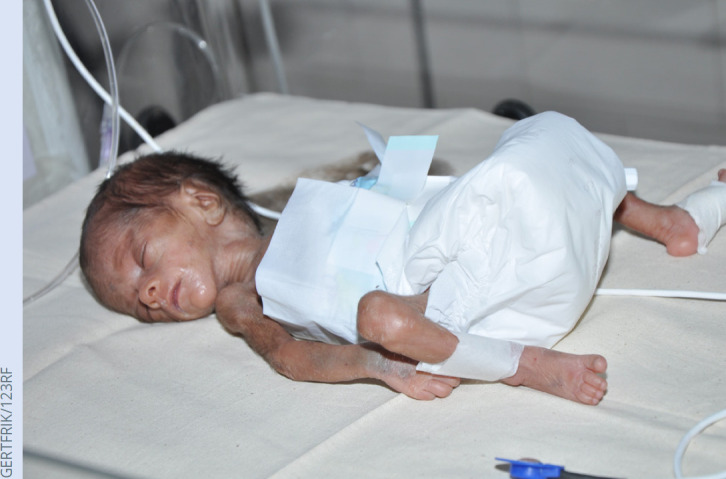

- The more preterm they are, the greater the risk
- Poor neonatal care increases the risk, even in less premature babies

## It is possible to prevent ROP from causing visual impairment and blindness. This requires:

**Figure:**
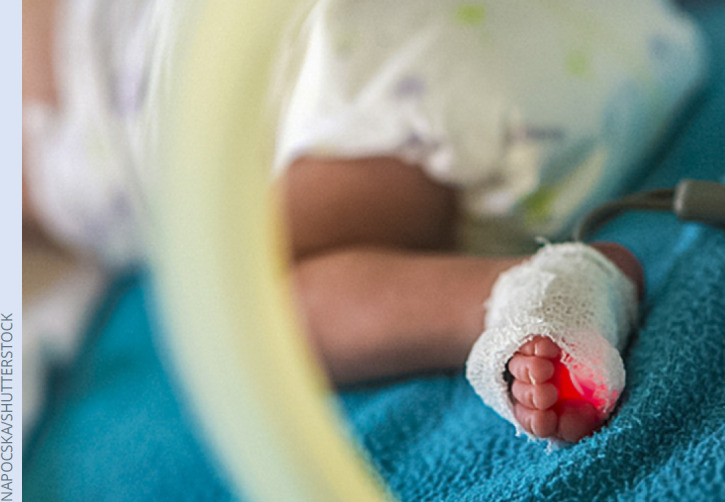

- High quality neonatal care. If there is not enough equipment to safely deliver and monitor oxygen, this must be strongly advocated for
- Screening: All babies at risk must be screened before 30 days after birth
- Treatment: Laser treatment should be given urgently, with confluent spots
- Follow-up: All children born preterm are at risk of visual impairment and must be followed up by an ophthalmologist and/or optometrist

## Parents are important members of the eye care and neonatal team

**Figure:**
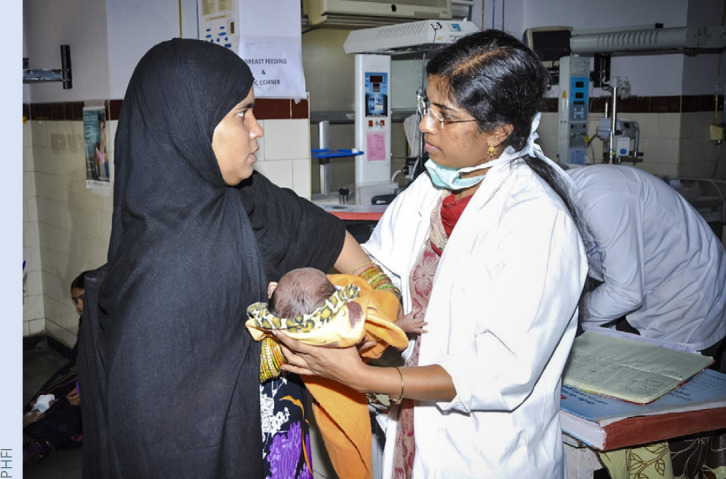

- Involve parents in the day-to-day care of the baby and encourage kangaroo care
- Keep parents informed of the need for screening and the results of screening, and the need for urgent treatment, if required
- Ensure parents understand the need for follow-up visits

